# Parent values and preferences underpinning treatment decision-making in poor-prognosis childhood cancer: a scoping review

**DOI:** 10.1186/s12887-022-03635-1

**Published:** 2022-10-14

**Authors:** Helen Pearson H, Gemma Bryan, Catherine Kayum, Faith Gibson, Anne-Sophie Darlington

**Affiliations:** 1 School of Health Sciences, University of Southampton, Southampton, UK; 2 The Oak Centre for Children and Young People, The Royal Marsden NHS Foundation Trust, Downs Road, Sutton, SM2 5PT Surrey UK; 3 School of Health Sciences, University of Surrey, Guildford, Surrey UK; 4 Member of the Parent and Carer Group, Patient Public Involvement, London, UK; 5 Centre for Outcomes and Experience Research in Children’s Health, Illness and Disability (ORCHID), Great Ormond Street Hospital for Children, NHS Foundation Trust, London, UK

**Keywords:** Cancer, Child, Decision-making, Parent, Poor-Prognosis

## Abstract

**Background:**

Parents of children who are diagnosed with a poor-prognosis cancer want to be involved in making treatment-related decisions for their child. They often make repeated decisions depending on their child’s response to treatment and can experience decisional regret as a consequence. Understanding parent values and preferences when making treatment-related decisions may help enhance discussions with healthcare professionals and identify additional ways of providing support to this parent population.

**Objectives:**

To explore parent values and preferences underpinning treatment decision-making for children receiving cancer-directed therapy for a poor prognosis cancer.

**Methods:**

A scoping review of research literature and systematic reviews from qualitative, quantitative, and mixed methods studies was conducted following Joanna Briggs Institute methodology. Articles which included parents of a child who received cancer-directed therapy for a poor-prognosis childhood cancer, under the age of eighteen years were considered. Four electronic databases were searched (CINAHL, Medline, PsychINFO, Web of Science Core Collections). Reference and citation lists of all included full-text articles were also searched. Summative content analysis was used to synthesise findings and develop themes.

**Results:**

Twelve articles were included. Parent decision-making was affected by underpinning factors: hope for a cure, fear of their child dying and uncertainty. Influencing factors: opinions of others, child’s wishes, and faith and religion had the potential to inform decision-making processes. Parents valued having enough time, being a good parent and being involved in decision-making. Preferences within these values varied resulting in the potential for conflict and ‘trade-offs’ in making decisions.

**Conclusions:**

Parent decision-making in poor-prognosis childhood cancer is complex and extends beyond values and preferences. Underpinning factors and values are consistent through the decision-making process with influencing factors and preferences varying between parents. Preferences can conflict when parents want to continue cancer-directed therapy whilst maintaining their child’s quality of life or can change depending on a parents’ cognitive state as they realise cure might be unlikely.

## Background

Approximately 400 000 children and young people aged 0–19 are diagnosed with cancer each year globally [1]. Of these, in the United Kingdom (UK), approximately 1,645 children are aged 0–14 when diagnosed [2]. In 2018, approximately 260 children died, accounting for 7% of childhood deaths in the 0–14 population that year [2]. Despite advances in research and treatment, there remains a small cluster of difficult to treat poor-prognosis childhood cancers. These include relapse neuroblastoma, medulloblastoma, hepatocellular carcinoma and peripheral T cell lymphoma.

Over the last decade, there has been an increase in clinical trials and treatment options for children diagnosed with poor-prognosis cancers, with the implementation of targeted inhibitors and immunotherapy treatments [3, 4]. Due to these options being available, parents often make multiple repeated treatment decisions as their child’s condition changes and relapses occur. Multiple relapses can often result in a poor-prognosis with an increased likelihood of death [5]. There is a need to understand individual parent values to support decision-making when their child has multiple cancer relapses [6].

### Treatment decision-making

Parents want to be involved in making treatment-related decisions [7]. Typically, parent involvement in decision-making happens when there is no standard of care treatment protocol, in situations where there is either disease progression, relapse, or failure to respond to treatment [8]. This situation is dependent on the diagnosis and the availability of standard of care protocols at these timepoints. Standard of care treatments, include those that are shown to be the best available for a particular disease, proven through clinical trials [9]. At this timepoint, treatment is guided by healthcare professionals based on the best available standard of care [10]. Parents are not involved in treatment decision-making at this point. Where there is no standard of care, parents can be offered various treatment options, they then become involved in making treatment-related decisions. Decision-making at this point will be influenced by parent values and preferences [10]. There is evidence showing how communication, support, having hope and focusing on their child’s quality of life were important considerations when making decisions [11]**.** However, this review did not focus specifically on parents’ whose child had a poor-prognosis cancer, where decision-making may differ due to the diagnosis and likely outcome. It cannot be assumed that values and preferences are the same for parents, regardless of their child’s outcome (survival, death, long-term disabilities as a result of diagnosis/treatment).

### Values and preferences

Treatment decision-making is informed by values and preferences which are underpinned by quality information on the risks and benefits of the treatment options, treatment expectations and cure [7]. Key components of quality information include diagnosis and treatment information, expectations of treatment, potential short and long-term side effects, and their child’s ongoing response to treatment [12]. This information needs to be explicit and provided by healthcare professionals in an open, accurate and clear way [11]. Research has shown the importance of clear prognostic information and the impact this can have on parent decision-making [13]. Understanding parent values and preferences can empower parents, increase their confidence in decision-making [14] and reduce the potential for decisional conflict and regret [15].

Research has acknowledged parents’ pursuit to continue cancer-directed therapies even when the chance of cure is minimal [16, 17]. Parent preferences for cancer-directed therapies in phase I clinical trials, or at the end-of-life, include the need to prolong life, minimise suffering [7], and know they have done everything possible to save their child’s life [16, 18]. Parents are reported to be overly hopeful and optimistic for the likelihood of cure which can result in parents making inadequate or inappropriate treatment decisions [12] which can impact on the child’s quality of life and suffering.

### Decisional regret

Decisional regret can have a lasting impact on parents particularly if their child dies [8.15,17]. One study found most parents regretted not pursuing further cancer-directed therapy or exploring alternative treatment options for their child [15]. Equally some parents regretted not discontinuing cancer-directed therapy earlier when this provided little or no benefit to their child [15]. This suggests conflict in parent treatment decision-making. Regret can result in the potential for prolonged grieving, be more intense and longer lasting, when compared with other types of grief [19].

Given parents want to be involved in making treatment decisions yet they experience decisional regret particularly when their child has a poor-prognosis cancer, this warrants exploration of the concepts of values and preferences which inform their treatment decision-making. Values and preferences are defined as the goals, expectations, predispositions, and beliefs an individual holds when making a decision [20]. Understanding these values and preferences could enhance discussions with healthcare professionals and identify additional ways of providing support to this parent population.

The parent population making treatment decisions for their child who has a poor-prognosis cancer is small. As a result, the research in this field is limited. Undertaking a scoping review allowed for a broad scope of the literature which provided identification of what is currently known relating to the concepts of parent values and preferences and how these inform treatment decision-making. To confirm a scoping review was the correct approach, the ‘what review is right for you tool?’ was completed (www.whatreviewisrightforyou.knowledgetranslation.net) [21].

The objective of this scoping review was to explore parent values and preferences underpinning treatment decision-making when their child was receiving cancer-directed therapy for a poor prognosis cancer. For this, there were two research questions: 1) what are parent values and preferences when their child is receiving cancer-directed therapy for a poor-prognosis cancer? 2) how do these values and preferences inform treatment decision-making?

## Methods

This review was conducted following the Joanna Briggs Institute (JBI) methodology [22]. For transparency, the objectives, methods, and inclusion criteria were specified in advance, and registered on 2^nd^ June 2020 with the Open Science Framework (https://osf.io/n7j9f). The protocol was published in BMJ Open [23].

The review process followed seven different stages: (a) defining the research questions, (b) eligibility criteria, (c) search strategy, (d) evidence screening and selection, (e) data extraction, (f) analysis of the evidence, and (g) presentation of the results [21]. Given the sensitivity of the scoping review topic, the authors included a consultation phase with a parent and carer group who have experienced making treatment decisions for their child with a poor-prognosis cancer. This mirrors the optional consultation phase suggested in the Arksey and O’Malley scoping review framework [24].

### Eligibility criteria

The eligibility criteria are outlined as per the JBI criteria (Table 1).

**Table 1:** Eligibility Criteria for this scoping review

Population	Parents of a child who had received cancer-directed therapy for a poor-prognosis cancer and the child was under the age of 18 years
Concept	Cancer-directed therapy was defined as any type of cancer treatment which could be received with or without palliative care or symptom management simultaneously Poor-prognosis relates to the cancer diagnosis and indicates whether the outcome is likely to result in death [9]. Poor-prognosis could be defined in anyway acknowledging that terminology differs between countries and clinicians
Context	Literature could encompass any clinical, medical or homecare setting Included was research literature and systematic reviews from qualitative, quantitative, and mixed methods studies

Articles were published in English, from any country with the full abstract available from 1996 until the time each database was searched.

### Search strategy

The databases included CINAHL, Medline and PsycINFO using the EBSCO platform and Web of Science Core Collections using the Clarivate platform. An initial search was conducted on 20^th^ April 2020 on CINHAL and Medline. This was followed by: Medline 6^th^ May 2021, CINAHL and PsychINFO 13^th^ May 2021 and Web of Science Core Collections 30^th^ May 2021. For completeness, the Web of Science was used to screen the reference and citation lists of all included full-text articles.

Keywords were searched for subject headings/Medical Subject Headings (MeSH) (Table 2). Phrases were not searched as these were natural language phrases for example “no realistic chance of cure” and “cancer directed-therapy”. Adjacency, truncation, and the wildcard search symbols provided full exploration of words. Boolean operators (AND/OR) were used. For combining keywords, subject headings, phrases, and MeSH terms the Boolean operator OR was also used. When combining the main topics (cancer, child, decision-making, parent, poor prognosis, values, and preferences) the Boolean operator AND was used. Full search strategies are shown in Table 3.

**Table 2:** Keywords and phrases searched

Keywords	Subject Headings/MeSH terms and phrases
Decision Making	“Decision making”
Cancer	Malignancy; Neoplasm; Oncology; “cancer-direct* therap*”
Child	Paediatric; Pediatric
Parent	mother*, father*, famil*
Poor Prognosis	Advanced Cancer; Deteriorate; Disease Progression; Incurable; Recurrent; Refractory; Relapse; Uncertainty; “advance* cancer”; “no realistic chance of cure”; “disease* progress*”; “palliat* chemotherap*”; “no reasonable chance of cure”
Value	Attitude; Belief; Choice; Choose; Expectation; Influence; Predisposition; Preference; Perception; “goal* of care”

**Table 3:** Full database search strategies

Database,Records Retrieved & Date Searched	Search Strategy
Medline Search Strategy (*N* = 218) Searched: 6^th^ May 2021	S1 parent* S2 mother* S3 father* S4 famil* S5 S1 OR S2 OR S3 OR S4 S6 value* S7 (MH "Value of Life") OR (MH "Social Values") S8 preference* S9 "goal* of care" S10 choice* S11 belief* S12 (MH "Social Norms") S13 attitude* S14 expect* S15 (MH "Motivation") S16 predisposition* S17 influenc* S18 experienc* S19 (MH "Clinical Decision-Making") S20 choose* S21 percept* S22 S6 OR S7 OR S8 OR S9 OR S10 OR S11 OR S12 OR S13 OR S14 OR S15 OR S16 OR S17 OR S18 OR S19 OR S20 OR S21 S23 "decision making" S24 (MH "Decision Making, Shared") S25 decision N2 making S26 decision* S27 decide* S28 S23 OR S24 OR S25 OR S26 OR S27 S29 S5 AND S22 AND S28 S30 "poor prognosis" S31 relaps* S32 (MH "Recurrence") S33 recurrenc* S34 refractory S35 incurable S36 (MH "Terminally Ill") S37 "advanc* cancer" S38 "no realistic chance of cure" S39 uncertain* S40 deteriorat* S41 (MH "Clinical Deterioration") S42 "disease* progress*" S43 "palliat* chemotherap*" S44 "no reasonable chance of cure" S45 S30 OR S31 OR S32 OR S33 OR S34 OR S35 OR S36 OR S37 OR S38 OR S39 OR S40 OR S41 OR S42 OR S43 OR S44 S46 child* S47 p#ediatric* S48 S46 OR S47 S49 cancer S50 neoplasm* S51 (MH "Neoplasm Recurrence, Local") S52 malignan* S53 (MH "Neoplasms") S54 "cancer direct* therap*" S55 oncolog* S56 S49 OR S50 OR S51 OR S52 OR S53 OR S54 OR S55 S57 S45 AND S48 AND S56 S58 S29 AND S57
CINAHL Search Strategy (*N* = 115) Searched: 13^th^ May 2021	S1 parent* S2 mother* S3 father* S4 famil* S5 S1 OR S2 OR S3 OR S4 S6 value* S7 (MH "Social Values") OR (MH "Values Clarification") S8 preference* S9 "goal* of care" S10 choice* S11 belief* S12 (MH "Health Beliefs") OR (MH "Attitude to Illness") S13 attitude* S14 expect* S15 predisposition* S16 influenc* S17 experienc* S18 choose* S19 percept* S20 S6 OR S7 OR S8 OR S9 OR S10 OR S11 OR S12 OR S13 OR S14 OR S15 OR S16 OR S17 OR S18 OR S19 S21 "decision making" S22 (MH "Decision Making, Shared") OR (MH "Decision Making, Clinical") S23 decision N2 making S24 decision* S25 decide* S26 S21 OR S22 OR S23 OR S24 OR S25 S27 S5 AND S20 AND S26 S28 "poor prognosis" S29 relaps* S30 (MH "Recurrence") S31 recurrenc* S32 refractory S33 incurable S34 "advanc* cancer" S35 "no realistic chance of cure" S36 uncertain* S37 deteriorat* S38 (MH "Clinical Deterioration") S39 "disease* progress*" S40 "palliat* chemotherap*" S41 "no reasonable chance of cure" S42 S28 OR S29 OR S31 OR S32 OR S33 OR S34 OR S35 OR S36 OR S37 OR S38 OR S39 OR S40 OR S41 S43 child* S44 p#ediatric S45 S43 OR S44 S46 cancer S47 neoplasm* S48 malignan* S49 "cancer direct* therap*" S50 oncolog* S51 S46 OR S47 OR S48 OR S49 OR S50 S52 S42 AND S45 AND S51 S53 S27 AND S52
APA PsycInfo Search Strategy (*N* = 91) Searched: 13^th^ May 2021	S1 parent* S2 mother* S3 father* S4 famil* S5 S1 OR S2 OR S3 OR S4 S6 value* S7 DE "Social Values" OR DE "Personal Values" S8 preference* S9 "goal* of care" S10 choice* S11 DE ‘’Uncertainty’’ S12 belief* S13 DE "Attitudes" S14 attitude* S15 DE "Parental Attitudes" OR DE "Parental Role" S16 expect* S17 predisposition* S18 influenc* S19 DE "Personal Values" OR DE "Social Influences" S20 experienc* S21 choose* S22 percept* S23 S6 OR S7 OR S8 OR S9 OR S10 OR S11 OR S12 OR S13 OR S14 OR S15 OR S16 OR S17 OR S18 OR S19 OR S20 OR S21 OR S22 S24 "decision making" S25 DE "Choice Behavior" S26 decision N2 making S27 decision* S28 decide* S29 S24 OR S25 OR S26 OR S27 OR S28 S30 S5 AND S23 AND S29 S31 "poor prognosis" S32 DE "Disease Progression" S33 relaps* S34 recurrenc* S35 refractory S36 incurable S37 "advanc* cancer" S38 DE "Terminal Cancer" S39 "no realistic chance of cure" S40 uncertain* S41 deteriorat* S42 "disease* progress*" S43 "palliat* chemotherap*" S44 "no reasonable chance of cure" S45 S31 OR S32 OR S33 OR S34 OR S35 OR S36 OR S37 OR S38 OR S39 OR S40 OR S41 OR S42 OR S43 OR S44 S46 child* S47 p#ediatric* S48 S46 OR S47 S49 cancer S50 DE "Neoplasms" S51 neoplasm* S52 DE "Oncology" S53 malignan* S54 DE "Neoplasms" OR DE "Oncology" S55 "cancer direct* therap*" S56 oncolog* S57 S49 OR S50 OR S51 OR S52 OR S53 OR S54 OR S55 OR S56 S58 S45 AND S48 AND S57 S59 S30 AND S58
Web of Science Core Collections Search Strategy (*N* = 149) Searched: 30^th^ May 2021	# 1 TOPIC: (parent* OR mother* OR father* OR famil*) # 2 TOPIC: (value* OR preference* OR "goal* of care" OR choice* OR belief* OR attitude* OR expect* OR predisposition* OR influenc* OR experienc* OR choose* OR percept*) # 3 TOPIC: ("decision making" OR decision NEAR/2 making OR decision* OR decide*) #4 TOPIC: #3 AND #2 AND #1 # 5 TOPIC: ("poor prognosis" OR relaps* OR recurrenc* OR refractory OR incurable OR "advanc* cancer" OR "no realistic chance of cure" OR uncertain* OR deteriorat* OR "disease* progress*" OR "palliat* chemotherap*" OR "no reasonable chance of cure") # 6 TOPIC: (child* OR p?ediatric*) # 7 TOPIC: (cancer OR neoplasm* OR malignan* OR "cancer direct* therap*" OR oncolog*) #8 TOPIC: #7 AND #6 AND #5 # 9 TOPIC: (#8 AND #4)

The University Research Librarian peer-reviewed the search strategy in each database for transparency and robustness. This was informed by the PRESS Statement [25]. No amendments to the search strategies were required.

### Evidence screening and selection

All articles were collated and uploaded into EndNote X9.2. Duplications were removed. The first level screen of title and abstract was completed independently by two reviewers (HP and ASD). Five articles had limited information provided within the abstracts. The third reviewer (FG) decided to include these articles for full-text review to avoid excluding potential research literature.

The second level screen involved full-text review. This was completed independently by the same two reviewers (HP and ASD). Each reviewer read the complete article and met virtually to discuss any conflicts. There were no discrepancies. The reference and citation list of included full-text articles were reviewed via the Web of Science by HP and resulted in additional articles for review. First and second level screening processes were repeated by HP and ASD independently, there were no discrepancies.

### Data extraction

Data were extracted into a table developed during the protocol phase and amended at this stage. Double data extraction was completed independently by two reviewers (HP and GB), a data extraction table was completed for each article. The reviewers met virtually to discuss each article and the data extracted. Data were duplicated in the extraction table therefore the table was amended. Amendments included merging ‘reasons why these cancer-directed therapies were chosen’ and ‘what parent values and preferences were identified’ as there was overlap in the data extracted for these questions. The citation details were extended to include the title of the article and journal. Table 4 shows the data extraction table. A third reviewer (FG) randomly selected 25% of the articles and completed the data extraction table as a quality check. The review team (HP, GB, FG) met virtually to discuss. There were no discrepancies.

**Table 4:** Amended data extraction table using in scoping review

Scoping Review Details	
Scoping Review Title	Parent values and preferences underpinning treatment decision-making in poor prognosis childhood cancer: A Scoping Review
Review Objectives	The objective is to explore parent values and preferences underpinning treatment decision-making when their child is receiving cancer-directed therapy for a poor-prognosis cancer
Review Questions	1) What are parent values and preferences when their child is receiving cancer-directed therapy for a poor-prognosis cancer? 2) How do these values and preferences inform treatment decision-making?
Inclusion/Exclusion Criteria (attributes)	
Population *(parent of a child under age of 18 years)*	
Concept *(parent of a child receiving cancer-directed therapy for a poor prognosis cancer)*	
Context *(hospital, community, hospice, other)*	
Types of Evidence Source *(Qual/Quant, mixed methods)*	
Evidence source Details and Characteristics	
Citation details *(first author, year of publication, journal, title of study, country of origin)*	
Study Aim	
Study Design (Prospective, Retrospective, Longitudinal or Cohort study)	
Research Methods	
Participant Details *(sample size, number of mothers, fathers)*	
Data extracted from sources of evidence	
Cancer-directed therapy in conjunction with palliative care/symptoms management	YES / NO / Not stated
What parent Values and preferences were identified *(Reasons why these cancer-directed therapies where chosen)*	
Explanation of how these values and preferences informed decision-making	
Article recommendations for future research	

### Patient public involvement consultation phase

Within the European paediatric oncology community, there is a move to include patient public involvement in all aspects of patient care [26] and involvement in the peer review publication process more widely [27]. The parent and carer group are a pre-existing Patient Public Involvement (PPI) group involved in a wider research study with the lead author (HP). One member (CK) of the group volunteered to be involved in reviewing the extracted data and work with the lead author to co-construct the results. This parent was given the extracted data from each included article on two specific questions: ‘what parent values and preferences were identified’ and ‘how did these values and preferences inform treatment decision-making’. This parent looked for recurring patterns in the data, considered the associated meaning and how these related to parent values and preferences in decision-making.

There is no consensus on how to approach consultation within scoping reviews [28]. The purpose of this consultation was to co-construct the results and write the manuscript to provide a true reflection of parent experiences. Engagement of this process was led by CK with meetings organised around CK’s availability allowing time between each discussion given the sensitivity of the topic and data content. Initial meetings were held virtually (due to covid19 restrictions) to discuss recurring patterns within the data. Parent experience and knowledge from clinical practice suggested the themes developed from the data interacted with each other and were not isolated in nature. We met face-to-face to discuss the themes and used visual diagrams to explore these relationships.

### Analysis of the evidence

Two authors (HP and CK) reviewed the extracted data relating to the research questions to look for recurring patterns in the literature. Recurring patterns which appeared similar were initially grouped as themes based on discussion between these two authors. To support and explore whether the themes identified were predominate in the literature, summative content analysis was conducted by one reviewer (HP) to identify repetitive words and phrases across the extracted data [29]. Summative content analysis searches for essential elements of text providing an entry point into the meaning of the whole data which forms discussions with co-researchers [30]. Co-researchers are involved in the analysis process with overall responsibility with the researcher [30]. This approach supported the integration of PPI involvement in the analysis of the extracted data supporting ongoing discussion of the recurring themes.

Repetitive words and phrases were initially grouped related to preferences and then the overarching value was considered. For example, the preference for more time with their child or time for the treatment to work resulted in the value, time. Underpinning, and influencing factors related to the unseen or unspoken effects on decision-making. These components of decision-making (values, preferences, underpinning and influencing factors) were seen to interact with each other however descriptively articulating these was difficult. The co-construction of the analysis between the authors (HP and CK) resulted in a map to visualise these different components. This approach to analysing extracted data ensured the analysis reflected the literature, related to the research questions, represented the parent experience, and provided opportunity to co-construct the interactions between the components of this complex decision-making.

Given the sensitivity of the scoping review topic, a patient and public consultation phase was included. This was a deviation from the a priori protocol [23].

### Presentation of the results

A total of 573 articles were identified via electronic databases (CINAHL = 115, MEDLINE = 218, PyscInfo = 91 Web of Science Core Collections = 149). Of these, 242 were duplicates leaving a potential 331 articles. Based on title, 305 articles were excluded, resulting in 26 articles for first level screening. Upon completion, 16 articles remained for full-text review.

At full-text review, a further six articles were excluded. One of the excluded articles was a mixed-method systematic review incorporating the facilitators and barriers to decision-making in non-curative childhood cancer [13]. This review consisted of eighteen articles, twelve of which were found in database searches. Five articles included in the mixed-method systematic review were excluded at first level screening (included in the 26 articles mentioned above), and seven articles were included in this review. For cross checking purposes, the remaining six articles in the systematic review were first level screened and deemed not relevant to the scoping review objectives. A total of ten articles were included from database searches.

The reference and citation list of these ten articles were searched. This yielded an additional seven articles. Two articles were excluded at first level screening. At full-text review, three articles were excluded and two were included. These articles were not identified through database searches due to missing keywords in the article title or abstract.

**Fig. 1:**
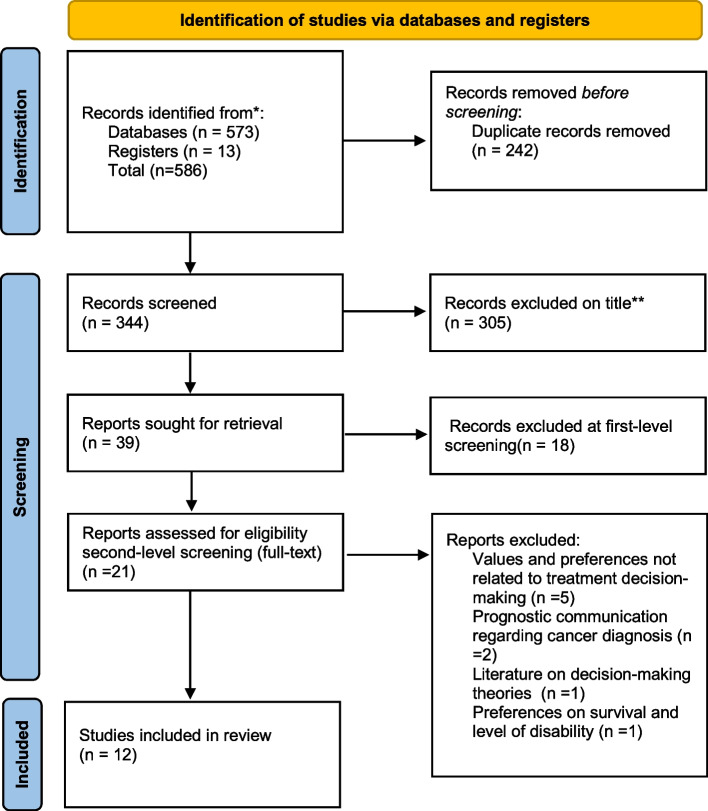
PRISMA Flow Diagram [31]

The PRISMA flow diagram (Fig. 1) details the final study selection and inclusion process. A total of 586 articles were identified, 573 through database searches, seven through the reference and citation lists of the final articles included from the database searches and six from the mixed-method systematic review [13] for cross-checking purposes. Once duplicates had been removed, 344 articles were screened. Three hundred and five articles were excluded based on title alone when the title did not reflect the scoping review objectives. For example, article titles relating to HPV vaccination, palliative care, end-of-life care, fertility preservation or healthcare professional perspectives were all excluded.

Thirty-nine articles were first level screened (twenty-six from database searches, six from the mixed-method systematic review and seven from the reference and citation lists of the final included articles from database searches). Eighteen were excluded at this stage (10 from database searches, six from the mixed-method systematic review and two from the reference and citation lists of the final included articles from the database searches).

Second level screening involved twenty-one articles (sixteen from database searches, five from the reference and citation lists of the final included studies). Nine were excluded at this stage. Twelve articles were included in this scoping review (ten from database searches and two from reference/citation lists).

### Description of the studies

The citation details of included articled can be found in Table 5. Most research was generated from the United States of America (US), represented by the first author for eight of the articles [8, 17, 32–37]. Research methods were stated for all studies with interviews being the primary source of data collection. Research design was not explicitly stated for each article. Eight of the studies were qualitative [8, 32–35, 37–39] and four were quantitative [16, 17, 36, 40]. One study was described as retrospective [8]. Other studies included bereaved parents but did not state a retrospective design. Three studies were described as prospective [17, 35, 38]. Studies had a significantly higher percentage of mothers participating than fathers, with one study having an equal number participating [16].

**Table 5:** Scoping review articles included in this review

First Author, Publication year	Title of Study	Journal	Country of Origin	Research Methods	Study Design	Sample Size	No of Mothers	No of Fathers	Study Aim	Authors Main Findings
Hinds, P.S. et al. (1996) [32]	Coming to Terms: Parents’ Response to a First Cancer Recurrence in their child	Nursing Research	USA	Semi-structured interviews, observations	Qualitative	33	27	5	Identify and describe the coping processes (meaning the behaviours and thoughts) used by parents of pediatric oncology patients to deal with stress of a first cancer recurrence in their child	“Coming to terms”: meant adapting to the situation, managing emotional reactions, making rational decisions in the hope for a cure whilst recognising their child may die. Concerns about treatment impacting on employment, finances, and family life. Fear of their child dying, hence fighting for a cure, searching for outcomes, consideration of child’s wishes, faith, and religion
Hinds, P.S. et al. (1997) [8]	Decision Making by Parents and Healthcare Professionals when Considering Continued Care for Pediatric Patients with Cancer	Oncology Nursing Forum	USA	Semi-structured interviews, questionnaires	Retrospective-descriptive design	39	Not stated	Not stated	To better define the treatment related decisions considered most difficult by parents of paediatric patients with cancer and the factors that influence their final decisions	Uncertainty of treatment side effects, ongoing communication from healthcare professionals on disease and treatment was vital, importance of trust and reassurance in healthcare professionals, consideration of their child’s wishes, repeated treatment decisions led to the conclusion that their child would not get better
Hinds, P.S. et al. (2000) [33]	An International Feasibility Study of Parental Decision Making in Pediatric Oncology	Oncology Nursing Forum	USA	Open-ended interviews	Qualitative Exploratory descriptive cross-sectional design	43	38	5	To describe parental decision making about treatment options for children with cancer and determine the feasibility of a similar but larger international study	Continuing with treatment was seen as doing everything possible to save child’s life, parents were satisfied if decisions made had a positive outcome, ongoing communication from healthcare professionals on disease and treatment was vital, consideration of child’s wishes, faith, and religion
Bluebond-Langner, M. et al. (2007) [35]	Understanding Parents’ Approaches to Care and Treatment of Children with Cancer when Standard Therapy has Failed	Journal of Clinical Oncology	USA	Semi-structured interviews, observation	Qualitative Prospective Ethnographic study	34	Not stated	Not stated	To examine US and UK parents’ approaches to care and treatment when standard therapy has failed and consider implications for clinical practice	Hope was leaving “no stone unturned”, seeking other treatment options, allowing time for treatments to work, for treatments to become available and more time with their child. Pursuing treatments whilst caring, protecting, and advocating for their child to prolong life, decrease suffering and keep all treatment options open
De Graves (2008) [38]	Lving with Hope and Fear – The Uncertainty of Childhood Cancer after Relapse	Cancer Nursing	Australia	In-depth interviews and field notes (case studies)	Qualitative Prospective Critical Ethnography	17	12	5	To develop an understanding of human actions and emotions that shape the experience of relapse, to question what influences the care provided at relapse, and to challenge current practice	Maintaining hope, pursuing treatments despite potential harm or suffering, exploring alternative therapies to reduce the fear of their child dying
Mack et al. (2008) [36]	Parents’ Views of Cancer-Directed Therapy for Children with No Realistic Chance of Cure	Journal of Clinical Oncology	USA	Close-ended questionnaires, vignettes	Quantitative	141	117	24	Assess parents’ experiences with treatment for their child with cancer and no realistic chance of cure	Hope for a cure, prolonging life and decreasing suffering
Hinds et al. (2009) [34]	“Trying to be a Good Parent” As defined by Interviews with Parents who made Phase I, Terminal Care and Resuscitation Decisions for their Child	Journal of Clinical Oncology	USA	Interviews	Qualitative Descriptive study	62	91.4%	44.8%	To define what it means to be a good parent to a child with incurable cancer	Ongoing communication from healthcare professionals on disease and treatment, being a “good parent” included making informed decisions in the best interests of their child
Maurer et al. (2010) [37]	Decision Making by Parents of children with Incurable Cancer who opt for enrolment on a Phase I trial compared with Choosing a Do Not Resuscitate/Terminal Care option	Journal of Clinical Oncology	USA	Interviews	Qualitative (secondary analysis)	62	53	9	To compare the self-reported rationale, good parent definition, and desired clinical staff behaviours of parents who recently decided for phase 1 (P1) chemotherapy with parents who chose do not resuscitate (DNR) or terminal care (TC) option	Importance of trust and reassurance in healthcare professionals, managing positive relationships with healthcare professionals, making informed decisions which were “evidence-based”, in the best interests of their child was seen as “doing the right thing”
Tomlinson et al. (2011a) [40]	Concordance between couples Reporting their Child’s Quality of Life and their Decision Making in Pediatric Oncology Palliative Care	Journal of Pediatric Oncology Nursing	Canada	Hypothetical scenarios interview, format	Quantitative Cross-sectional study	26	13	13	To (1) describe concordance between fathers' and mothers' evaluation of QoL and (2) determine correlation between mother and father for how factors such as hope, anticipated QoL, and prolonged survival time influence the decision between supportive care alone versus aggressive chemotherapy	Maintaining hope outweighed their child’s quality of life and suffering, opinions of family members, other parents and healthcare professionals was influential, treatment provided more time with their child
Tomlinson et al. (2011b) [16]	Chemotherapy versus supportive Care alone in pediatric palliative care for cancer: comparing the preferences of parents and health care professionals	Canadian Medical Association Journal	Canada	Hypothetical interviews (prepared scripts, visual aids)	Quantitative	77	60	17	The goal of this study was to compare the strength of preference between parents and healthcare professionals for supportive care alone versus palliative chemotherapy for children whose cancer has no reasonable chance of being cured, and to determine how specific factors affect these preferences	The importance of hope outweighed their child’s quality of life and suffering, opinions of family members, other parents and healthcare professionals was influential, treatment provided more time with their child
Matsuoka et al. (2012) [18, 39]	Parents’ thoughts and perceptions on hearing that their child has incurable cancer	Journal of Palliative Medicine	Japan	Semi-structured open-ended interviews	Qualitative	23	16	7	To describe parental thoughts and perceptions when they hear that their child has incurable cancer	Continuing treatment was to not give up on their child, recognising death was not a potential reality with the focus on not allowing their child to die. This outweighed their child’s quality of life and suffering whilst acknowledging their child’s struggle to tolerate treatments
Mack et al. (2019) [17]	Unrealistic Parental Expectations for Cure in Poor-Prognosis Childhood Cancer	Cancer	USA	Questionnaires	Quantitative Prospective cohort study	95	81%	19%	The goal of the current study was to identify communication and care experiences that drive parents' care goals and decisions	Focus was to cure their child, allowing time for treatments to work and for treatments to become available

Poor-prognosis was defined in several ways using different terminology. Studies included parents whose child had previously died from cancer [8], where there was disease progression [33, 35], recurrence [8, 33, 35], relapse [38], or the child was at a terminal stage [33]. In these situations, the probability of cure will have decreased resulting in a poor-prognosis compared with initial treatment at diagnosis. In other studies poor-prognosis was defined as ‘no realistic chance of cure’ [36], < 5% chance of long-term survival [16, 40], making a noncurative treatment decision [34], incurable cancer as defined by the child’s healthcare team [37, 39], or statistical data related to the specific disease type [17].

### What informs treatment decision-making?

**Fig. 2:**
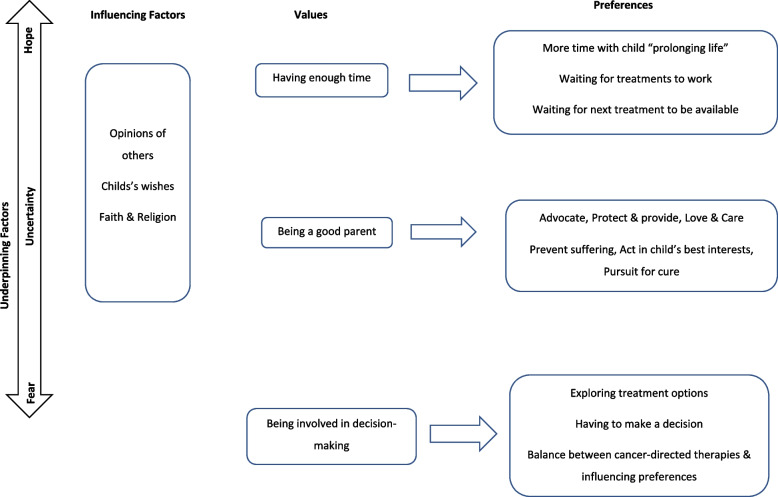
Results map on what informs parent treatment decision-making in poor-prognosis childhood cancer

Underpinning parent drive to continue with cancer-directed therapy were three factors: hope for a cure, fear of their child dying and uncertainty. These factors were constant through parent treatment decision-making, underpinning decisions which were made. Aspects which influenced parent decision-making included the opinion of others, child’s wishes and faith and religion. Parents valued having enough time, being a good parent and being involved in the decision-making process. Within these values were varying preferences which at times were conflicted and involved ‘trade-offs’ in order to pursue and continue with cancer-directed therapy. Figure 2 maps how underpinning factors, influencing factors and values and preferences informed parent treatment decision-making.

### Underpinning factors

#### Hope for a cure

Hope was fundamental in the pursuit for cancer-directed therapies. When informed of a new relapse parents underwent a process of “coming to terms”, adapting to what was happening to their child [32]. This adaptation involved parents’ managing their emotional reactions to make rational decisions in the hope for cure with the conflicting lingering possibility that their child might die [32]. Hope for a cure encouraged parents to believe this was possible and parents searched for this in conversations with healthcare professionals [38]. For parents, maintaining hope was the driving force in pursuing treatment and in some cases outweighed their child’s potential harm, suffering [38] and quality of life [16]. Continuing cancer-directed therapy in some cases was a parent obligation which corresponded with being a good parent and represented not giving up [39]. A chance for survival provided hope [33] and hope was ranked highest in the need to continue cancer-directed therapy [40]. One study found disagreement between mothers and fathers relating to quality of life and hope resulting in gender differences to continuing cancer-directed therapy [16].

Hope could be associated with leaving “no stone unturned” [35]. Despite parents recognising a cure was unlikely, parents continued to hope for a cure and therefore continued with cancer-directed therapy [36]. Treatment options included exploring alternative therapies [38] and seeking treatment opinions outside of what their child’s healthcare team offered [35]. The obligation to continue treatment, despite the knowledge that cure was unlikely resulted in parents pursuing treatments to ensure they had done everything possible to save their child’s life [33], in effect leaving “no stone unturned” [35]. This related with being a good parent, to keep all options open and not allow their child to die despite knowledge that this was a potential.

#### Fear of their child dying

Exploring second opinions and alternative therapies were all strategies in managing fear of their child dying, to reduce the potential of that becoming a reality [38]. This was what a good parent would do and in doing so this maintained hope in finding a cure.

#### Uncertainty

Uncertainty related to the unknown of how their child would tolerate treatment and the treatment outcome. Parents considered the effects of treatment on their child [8], and if treatment provided a positive outcome, then parents were satisfied with the decisions which they had made [33].

Adjustments within the family unit and parent role caused uncertainty. Parents were concerned how treatment would impact their employment, finances, and family life whilst simultaneously fearing their child would die with losing a sense of normality [32].

The uncertainty of their child’s outcome provided a “cognitive shift” between cure and death resulting in “fighting for cure” and “preparing for loss” [32]. The need to pursue all treatment options available, being a good parent maintaining hope and enable time with their child. This ensured parents had done everything possible whilst acknowledging treatment might not work, and death was a possibility. Not all parents were open or willing to consider this cognitive shift and did not recognise death as a potential reality [39].

### Influencing factors

#### Opinions of others

The opinions of family members, other parents and healthcare professionals influenced parent decisions for cancer-directed therapy [16]. Searching outcomes from families that had gone through treatment and whose child had died helped parents in their adjustments [32].

The actions, information, communication, and support from healthcare professionals were major factors in parents feeling they were being accepted in pursuing cancer-directed therapies. Open communication on their child’s clinical condition, treatment response [8, 33, 34] and knowing everything possible was being done provided a sense of trust and reassurance [8, 34, 37]. A positive relationship where healthcare professionals knew the family and child provided a sense of belonging [34, 37].

#### Child’s wishes

The child’s wishes were acknowledged in some studies [8, 32, 33]. This related to children expressing the extent of their symptoms and their preferences around the continuation of treatment.

#### Faith and religion

Faith and religion were important factors from studies particularly in the US [32, 33, 40]. This related to being guided by religion and beliefs that their child’s outcome was out of their control.

### Values and preferences

#### Having enough time

Parents valued the need for time and was a component as to why parents choose to continue with cancer-directed therapies. Time related to more time with their child, “prolonging life”, to give time for treatments to work or time for treatments to become available, time to realise and adjust to the situation and time to make informed decisions [16, 17, 33, 35].

Specific to buying time was parents wanting to start new treatments earlier or giving them longer time to work which enabled more time with their child [35]. Informed decisions having explored all possible treatment options enabled parents to plan for future possibilities for their child [33]. Parents valued time and the preferences of what time could give them.

#### Being a good parent

A good parent to a sick child pursued cancer-directed therapies, cared, protected, and advocated for their child within the healthcare professional team [35]. The definition of a “good parent” to a child with incurable cancer [34] was defined as unconditional love, provided basic provisions such as a home, clothing and food, prevented suffering, provided protection, promoted health, was a life teacher and made unselfish informed decisions in the best interests of their child [34, 37]. A good parent continued cancer-directed therapy to cure [17, 39], keep all treatment options open [35], and did not allow their child to die despite realisations that this could be the outcome [39].

#### Being involved in decision-making

Parents valued being involved in the decision-making process. Decision-making included parents exploring treatment options and navigating the options available to them. In some circumstances, parents felt they did not have a choice if they wanted their child to live with the need to “fight for life” [28]. Parents made decisions which were evidence-based [33, 37], yet experienced turmoil between fighting for a cure and having to decide [32]. There was strong emphasis on the need to continue cancer-directed therapies [35, 37, 39], yet this need in some circumstances was weighed against their child’s quality of life, level of suffering, others’ opinions [16, 39, 40], child’s wishes and religious beliefs [33]. Parents tried to balance saving their child with cancer-directed therapies and protecting from suffering and harm [38]. In the process of deciding, some parents were influenced by more than one preference [33] which had the potential to cause conflict within their decision-making. There was a sense of needing to “do the right thing” [33, 37] however a definition of what this meant was lacking.

In some circumstances, parents made repeated decisions relating to whether to continue cancer-directed therapy [8]. This was dependent on their child’s response to treatment and toxicities experienced. During the process of “coming to terms”, parents began to realise their child’s ability to tolerate and respond to treatments, the suffering this was causing and therefore started to consider the limitations of continuing treatment [32]. Despite literature which suggests parents may reach this realisation, this does not necessarily mean parents will discontinue cancer-directed therapy. Parents continued cancer-directed therapy for goals other than cure such as prolonging life and to decrease suffering [35, 36]. Awareness that their child was deteriorating with the potential for their child to struggle to tolerate further treatments and subsequently die was acknowledged [32, 39]. Parents recognised there could be an endpoint to cancer-directed therapy, and they might have to participate in making that decision [32]. For some parents, the need to repeatedly make decisions lead to the conclusion that their child would not get better resulting in the final decision to terminate cancer-directed therapy [8].

## Discussion

Childhood cancer diagnoses in the UK is considered rare [2]. Parents whose child has a poor-prognosis cancer remains a smaller cluster within this. This parent population is often under-researched, it being a small sub-set within a rare disease and the ethical implications of involving parents in research of this nature. Nevertheless, as researchers and healthcare professionals it is our duty to foster ways to address the needs of this parent population who are making complex, difficult treatment decisions for their child in a state of high emotion. This scoping review included twelve articles. Knowledge is informed by a total of 590 parents, predominately represented by the voice and experiences of mothers (*N*-413) with the fathers’ voice continuing to be underrepresented (*N* = 108). Two studies did not provide a gender breakdown [8, 35]. Only one study included parents from the UK [35]. With different healthcare systems internationally, access to treatments is fragmented resulting in the navigation of treatment decision-making for parents being difficult and uncertain. As a result, treatment decision-making is non-linear, with great complexity and is highly emotional for parents.

The complexity of parent treatment decision-making resulted in three aspects: underpinning factors, influencing factors and values and preferences. Underpinning factors were constant through the decision-making process. These could be interrupted as parent psychological and emotional aspects within decision-making. Influencing factors were those parents may consider when making treatment decisions but appeared to hold different weighting to parents across the data. Parents valued having enough time, being a good parent and being involved in decision-making. Each of these values contained preferences, aspects which were important to parents yet ones they might be willing to compromise on in order to continue with cancer-directed therapy. The literature defines these as ‘trade-offs’, the risks and benefits associated with each treatment option [41] and what parents were willing to accept in relation to their values.

Parents acknowledged their child may die, yet this did not result in a linear process of moving from cancer-directed therapy to symptom management/palliative care alone. The preferences of continuing cancer-directed therapy could alter at this timepoint with the overriding need to maintain hope, have time with their child and leave “no stone unturned” [35, 36]. This timepoint could be defined as a ‘tipping point’ in identifying a change of goals from continuing cancer-directed therapy in the hope of a cure to the realisation that their child will not survive and wanting to increase time with their child.

Parents experienced emotional adjustment when their child’s cancer relapsed managing the grief, shock, and anguish [32]. Parent emotions may fluctuate throughout the decision-making process depending on their child’s clinical condition, the decisions to be made and their own adjustment to the situation. Emotions have the potential to influence rational decision-making processes [32]. There is a body of decision-making literature on how people make decisions which is typically divided into rational [42, 43], descriptive/psychological [44, 45] and emotional [46] decision-making. Rationally, parents want to make informed decisions opting for the best treatment that is underpinned by science and statistics. Descriptive/psychological decision-making acknowledges how parents may obtain and process information, and how their previous experiences and intuition can influence decision-making. Emotion in decision-making focuses on parent feelings and emotions of the situation which can be informed by previous experiences, their current situation and anticipated future emotions [46]. The combination of these three decision-making components, (rational, emotional, and descriptive) may produce conflict in the pursuit of a decision that is underpinned by parent values and preferences and in the best interests of their child.

The “coming to terms” between cure and death was not seen in all parents which suggests hope as a central underpinning factor to these parents whose focus was solely on cure [32]. Parents may subconsciously acknowledge what is happening but doing so consciously means confronting or speaking about this fear which some parents may not be able or willing to do. The value of being a good parent and the preferences associated with this differed between parents. Preferences appeared conflicted wanting to prevent suffering, protect their child and act in their child’s best interests whilst continuing with cancer-directed therapies [34, 37] which could induce suffering and not be in their child’s best interests. Parents emphasised quality of life and to reduce suffering and harm [40]. However, only one study explicitly stated symptom management was given in conjunction with cancer-directed therapy [35]. It cannot be assumed that parents did not engage with symptom management, more that studies did not clearly identify this. A good parent continued cancer-directed therapy to cure even when parents realised their child may die. The need to continue could be underpinned by hope. Hope for a cure enabled parents to pursue cancer-directed therapy even at the expense of the child’s suffering.

The involvement of the child’s wishes is likely to be dependent on age and cognitive ability. Protection was a core component of being a good parent [34] and in some instances parents my protect their child from the full extent of the seriousness of the situation.

In the early 2000s the internet and use of social media platforms became more established. Nowadays many childhood cancer diagnoses have parent-led social media pages on platforms such as Facebook, Instagram and Twitter with a community that share personal knowledge and experience. Furthermore, parents can research treatment options and clinical trials internationally empowering them to explore treatment options beyond what their child’s clinician offers which was seen in some studies [35, 38].

Parents are faced with making complex treatment decisions when their child has a poor-prognosis childhood cancer. Their values can contain conflicting preferences in opting for cancer-directed therapy. Research has shown regret is less evident in parents who trust their child’s oncologist, have concise prognostic information, and are involved in the decision-making process [7]. This reinforces the need to provide adequate support in the decision-making process. Treatment decision-making becomes complex when there is no standard of care treatment protocol resulting in the potential for parents to make multiple repeated treatment decisions as their child’s condition changes. Understanding these parent values and preferences and how they inform treatment decision-making provides the basis for developing support tools such as decision aids to support parent treatment decision-making and enhance discussions between parents and healthcare professionals.

Intervention processes such as the Medical Research Council for developing complex interventions [47, 48] could support the development of support tools for use in clinical practice. The use of co-design and co-production methods provide a cohesive opportunity for developing interventions [49, 50]. In complex decision-making these methods, working in partnership with stakeholders would ensure the intervention meets the needs of parents whilst being underpinned by empirical research.

### Patient public involvement consultation discussion

The parent and carer group reviewed the study results to provide input into how representative these were to parent experiences. The definition of values and preferences resonated with parents, but the individual meaning was seen as subjective to each parent. For example, what one parent may define as suffering another parent may not. The need for parents to define their meaning of a preference may support decision-making discussions with healthcare professionals in clinical practice. The group saw preferences as the trade-offs parents made to prioritise what they valued most. For example, one parent spoke of prioritising prolonging life but in doing so this may increase suffering resulting in conflicting preferences.

The complexity of decision-making was hard to define. For example, the emotion of decision-making was lacking, and the literature was ‘sanitised’ as a definitive way of how parents made decisions but in reality, it is not linear and difficult to articulate. One study alluded to the emotional component of making treatment decisions [32]. Parent emotion is huge in decision-making and one that the group felt was not fully addressed within the literature.

The values presented were an accurate perception of decision-making in poor-prognosis childhood cancer, but the richness and depth of the complexities of this decision-making was not captured possibly due to the topic sensitivity. The group acknowledged limitations for example journal word restrictions, or the data collection methods used which could inhibit the understanding of these complexities. For example, the use of questionnaires for a sensitive topic felt inappropriate and data produced during interviews is dependent on the trust built between parent and researcher particularly if there is no prior relationship.

The group spoke of a constant shift in parent values and preferences relevant to the situation. How a parent defines these values and preferences is not constant through time but changes as the situation develops depending on their child’s clinical condition and treatment decisions to be made. This was acknowledged in a shared decision-making framework where values and preferences are not seen as stable, but change based on parent capacity and reflection [51]. The group represented this shift as a weighing scale, the options parents have at each decision point with the compromises (preferences) required to make that decision. Parents spoke of this shift being underpinned by a constant feeling of pressure, needing to do the right thing for their child, not wanting to let their child down and the uncertainty and fear that their child may or is dying. This related to the underpinning factors of hope for a cure, fear of their child dying and uncertainty.

The language used within the literature caused concern. Words such as “fighting” were seen in a negative context like a parent going to war. The group had experienced parents using words like fight, battle, winning and losing in clinical practice. This language does not support parents in their “cognitive shift” and adjustment to the situation.

Parents would regret their decisions if their child had died as an outcome, was the consensus of decisional regret. As a result, retrospective study designs could produce biased findings and not be a true representation of parent decision-making at the time of making those decisions. A more accurate representation would involve parents at or just after the time of making a decision whilst the decision outcome is unknown. The group felt parents would value prospective study designs which provide opportunity to talk through their decision-making processes in real time. They did not feel strongly that this approach would add burden to parents.

The group discussed support ‘tools’ and the development of an intervention which supports parent decision-making in clinical practice. Consensus within the group was that an intervention would need to acknowledge that parent values and preferences, underpinning and influencing factors in decision-making are individual and shift in response to their child’s treatment and clinical condition. The purpose of any intervention would be to clarify and organise parent thoughts, provide a ‘spring-board’ to decision-making which provided additional aspects to consider and act as an enabler to start conversations with significant others including healthcare professionals about what is important to them when making treatment decisions.

### Limitations

This scoping review only included studies written in English due to financial constraints of translating this literature. Grey literature was not included as per the a priori protocol [23]. Having these two exclusions may have missed literature relating to this topic.

One study included parents whose child was between 1–24 years old at the time of death [8]. This study did not provide details on how old the child was at the time of receiving treatment nor were the results broken down by age groups. Although all relevant data were extracted it is not known whether this was relevant to those under the age of 18 years.

There was a lack of father involvement across the studies. There is a need to increase the participation of fathers in research such as this to further explore whether there are gender differences in the values and preferences which underpin treatment decision-making. One research study suggested there were differences [16].The literature on decision-making acknowledges rational, descriptive, and emotional processes in decision-making. The parent and carer group provided consensus that parent emotion in treatment decision-making had not been addressed fully in the literature. Emotion can be difficult to articulate which might be the reason why there is limited acknowledgement of this in the literature.

## Conclusions

The complexity of treatment decision-making in poor-prognosis childhood cancer considers more than parent values and preferences. There are constant underpinning factors to these decisions for parents, hope for a cure, fear of their child dying and uncertainty throughout the decision-making process. Influencing factors can support or refute the treatment options parents are considering yet these are not consistent factors for every parent. Values appeared consistent but the preferences within these can be conflicting resulting in complex decision-making which is in their child’s best interests. Preferences conflict when parents want to continue cancer-directed therapy whilst maintaining their child’s quality of life or can change depending on a parents’ cognitive state as they realise cure might be unlikely. Complexities of decision-making in this situation is difficult to articulate and the literature lacked the emotional component involved in making these decisions.

### Implications of the findings

Healthcare professionals working in clinical practice need to consider the investment of additional time with parents to explore what is important to them when making treatment decisions for their child. Allowing time to communicate effectively will help promote parent inclusion, confidence, and clarity in the treatment decisions they make. Furthermore, this could be supported by digital interventions such as support ‘tools’ which help parents clarify their decision-making and assist in conversations with healthcare professionals.

The findings acknowledged ‘the opinions of others’ as an influencing factor in parent decision-making. It is important to recognise those involved and contribute to the decisions parents make can incorporate a range of people including healthcare professionals, extended family, friends, siblings and other patients.

Further research in the exploration of emotion in this type of decision-making is warranted. Preferences in treatment decision-making can change depending on several aspects. Research to explore how these values and preferences may change over time with parents who make multiple repeated treatment decisions could provide more effective ways of supporting parents in clinical practice. Studies included in this review stated the need to develop decision support tools, guidelines and care models which can be integrated into clinical practice. Development of such tools and models are required to support parents in clinical practice in this complex decision-making.

## Data Availability

Data extracted using the data extraction tool are available from the corresponding author on reasonable request. As this is not empirical data owned by the authors the data extracted is available within the public domain.

## Abbreviations

CINAHL: Cumulative index of nursing and allied health research
JBI: Joanna briggs institute
MeSH: Medical subject headings
PRISMA: Preferred reporting items for systematic reviews and meta-analysis’
UK: United kingdom
No: Number
US: United States of America
